# Highly photosensitive graphene field-effect transistor with optical memory function

**DOI:** 10.1038/srep15491

**Published:** 2015-10-20

**Authors:** Shohei Ishida, Yuki Anno, Masato Takeuchi, Masaya Matsuoka, Kuniharu Takei, Takayuki Arie, Seiji Akita

**Affiliations:** 1 Department of Physics and Electronics, Osaka Prefecture University, 1-1 Gakuen-cho, Naka-ku, Sakai, Osaka 599-8531, Japan; 2 Department of Applied Chemistry, Osaka Prefecture University, 1-1 Gakuen-cho, Naka-ku, Sakai, Osaka 599-8531, Japan

## Abstract

Graphene is a promising material for use in photodetectors for the ultrawide wavelength region: from ultraviolet to terahertz. Nevertheless, only the 2.3% light absorption of monolayer graphene and fast recombination time of photo-excited charge restrict its sensitivity. To enhance the photosensitivity, hybridization of photosensitive material and graphene has been widely studied, where the accumulated photo-excited charge adjacent to the graphene channel modifies the Fermi level of graphene. However, the charge accumulation process slows the response to around a few tens of seconds to minutes. In contrast, a charge accumulation at the contact would induce the efficient light-induced modification of the contact resistance, which would enhance its photosensitivity. Herein, we demonstrate a highly photosensitive graphene field-effect transistor with noise-equivalent power of ~3 × 10^−15^ W/Hz^1/2^ and with response time within milliseconds at room temperature, where the Au oxide on Au electrodes modulates the contact resistance because of the light-assisted relaxation of the trapped charge at the contact. Additionally, this light-induced relaxation imparts an optical memory function with retention time of ~5 s. These findings are expected to open avenues to realization of graphene photodetectors with high sensitivity toward single photon detection with optical memory function.

Image-sensing devices such as charge-coupled devices (CCDs) have become indispensable not only for daily life but also for industrial applications. The charge accumulation induced by incident photons to the device is a crucially important aspect of imaging devices with sufficiently high sensitivity for single-photon detection. Monolayer graphene has been predicted theoretically to have uniform light absorption at ultra-wideband wavelengths^1^. Consequently, graphene is anticipated for use in optical sensors^2^ with an ultra-wide wavelength region extending from ultraviolet to terahertz with high-speed photoresponse^3^,^4^. That high sensitivity is attributable to graphene’s zero-gap band structure and unusually high mobility^5^. Even given these benefits, the photogenerated electron–hole pairs in graphene normally recombine within a few tens of picoseconds^6^,^7^, which results in the poor intrinsic photoresponse of the graphene photodetectors of ~1 × 10^−2^ A/W. One strategy to improve photosensitivity is “preventing recombination” or “enhancing the charge separation of photo-excited hole-electron pairs”. The photocurrent is expected to be enhanced if an additional external field is applied to separate the photogenerated holes and electrons. A strong electric field at regions adjacent to the metal electrodes to graphene channel engenders efficient photocurrent generation^3^,^4^,^8^,^9^,^10^ resulting in >30% efficiency for electron−hole separation, as revealed using scanning optical microscopy.

Further enhancement of photosensitivity was achieved by introducing a charge accumulation element such as a trapping site adjacent to the channel of a graphene field-effect-transistor (G-FET). The photogenerated carriers in a graphene modulate the distribution or density of accumulated charges, consequently modulating the conductance of the channel through a gating effect^11^,^12^,^13^. A similar strategy for enhancing photosensitivity is hybridization: a combination of the material for the photoinduced carrier generation and the graphene as a charge-sensing element for the photogenerated carrier^14^,^15^,^16^. In addition to the enhancement of photosensitivity, multifunctional photoresponsive memory devices using graphene-MoS_2_ hybrid structures have been reported^15^. They show extremely high photosensitivity of 5 × 10^8^ A/W and gate-tunable persistent photoconductivity induced by the trapped charges. Charge accumulation is necessary to realize high sensitivity in graphene photodetectors, either with or without hybridization of other materials. In these cases, however, using charge accumulation for gating the graphene channel produces a slow response of around seconds to minutes, which is necessary for large-quantity charge accumulation to induce efficient gating^11^,^12^,^13^,^14^,^15^,^16^.

It is noteworthy that the current flow through the G-FET channel can be modified not only by the gating effect but also by electrical contacts to the graphene channel as source and drain electrodes in G-FET. Great efforts have been devoted to improving the electrical contact to the graphene because the contact resistance strongly restricts the G-FET performance^8^,^10^,^17^,^18^,^19^,^20^,^21^,^22^,^23^,^24^,^25^. The channel conductance of G-FET is modulated if the accumulated charge near the contact can be controlled by the light irradiation because of the barrier height modification. Although gold is a common material for use in electrodes, a thin oxide layer of Au would be formed on the electrode surface. Very recently, the efficient charge transfer from AuO_x_ layer with thickness of ~20 nm to graphene under light illumination has been demonstrated, where the AuO_x_ layer behaves as the photoinduced carrier generation layer^26^. This result implies that the thin AuO_x_ layer on the contact electrodes acts to modify the contact resistance under light illumination.

This report describes that G-FET with a thin barrier layer of AuO_x_ layer at the contact presented in Fig. 1a exhibits high photosensitivity of ~6.1 × 10^4^ A/W (~25 pA/photon) at room temperature and optical memory function, where the Au oxide layer on the Au electrodes acts as photoinduced variable contact resistance.

Figure 1a presents a schematic illustration of photosensitive G-FET with a thin barrier at the source and drain electrodes, where the graphene was on the Au electrodes. Generally, graphene is transferred before the fabrication of source and drain electrodes to ensure good contact between them. Here, we transfer the graphene on the source and drain Au electrodes after the formation of a thin barrier on the electrodes. Source and drain electrodes consisting of Cr/Au (5 nm/30 nm) were fabricated on a highly doped Si substrate with a 300 nm-thick SiO_2_ layer. Subsequently, the Au electrodes were oxidized by O_2_ plasma with RF (100 kHz) power of 100 W for 60 s to form the Au oxide layer on the electrodes. X-ray photoelectron spectroscopy (XPS) was performed using Mg-Kα radiation (1253.6 eV), where the binding energy was corrected using the C1s peak at 284.6 eV. After the Au-electrode oxidation, a monolayer graphene was transferred onto the substrate and was trimmed using oxygen plasma etching to form an FET channel (width × length: 5 × 2 and 2 × 4 μm), where the graphene was synthesized using low-pressure chemical vapor deposition at 1000 °C using Cu foil as catalyst^27^,^28^ (see Fig. S1 for Raman spectrum of grown graphene). All electrical measurements were taken in vacuum at ~10^−3^ Pa.

Figure 1b,c show X-ray photoelectron spectroscopy (XPS) spectra for Au 4f before and after the plasma oxidation processes. Chemical shifts of Au 4f peaks induced by the oxidation process are observed clearly to be ~1.2 eV, which are determined to be oxidized Au^29^. Figure 1d presents transfer characteristics of G-FET with plasma oxidized electrodes measured under dark and illuminated (510 nm, ~ 228 μW/cm^2^) conditions at room temperature, where collimated light with a beam diameter larger than ~20 mm through a color filter was used as the light source. At gate voltage (*V*_GS_) lower than 20 V, transfer characteristics measured at both the dark and illumination conditions were almost identical, where the peak field effect mobility was ~600 cm^2^/Vs. At *V*_GS_ > 20 V with a *V*_GS_ sweep rate of 14 V/s, the photoresponse of the source–drain current *I*_*DS*_ appeared as presented in Fig. 1d. It is noteworthy that the G-FET of which the graphene channel was transferred immediately after the fabrication of electrodes without oxidation showed no remarkable photosensitivity (see Fig. S2). Figure 1e exhibits the difference of *I*_DS_ measured in dark and illumination conditions (Δ*I*_DS_). The Δ*I*_DS_, either negative or positive, depends on the sweep rate of *V*_GS_; it increases to 1.5 μA (6.5 × 10^4^ A/W) with increase of the sweep rate (see also Figs 3S and S4). A possible scenario to explain this photoresponse is that the certain charge accumulation (trapping) sites^11^,^12^,^13^, which contribute to the photoresponse of the G-FET, were created by the O_2_ plasma treatment. Despite the high photosensitivity of 6.5 × 10^4^ A/W, the continuous shift of *V*_Dirac_ was observed for the repeated cycle of the measurements because of the pileup of the trapped charge and was returned slightly to the initial *V*_Dirac_. Consequently, the plasma oxidation induces the strong charge trapping site on SiO_2_ in addition to formation of the Au oxide layer on the source and drain electrodes.

To reduce the damage to the SiO_2_ layer induced by the oxidation process, we performed a more moderate oxidation process as natural oxidation of the Au electrodes. The substrate with the as-deposited Au electrodes was left intentionally in air for a few months to form a native oxide layer with less damage to the SiO_2_ layer. Figure 2a shows that the Au oxide related peak shifts in XPS spectra are visible after natural oxidation. The peak shifts are much smaller than those for the plasma oxidation, although the intensities are higher than those for the plasma oxidation, which indicates that the oxidized Au formed by the natural oxidation process has smaller valence of Au than that formed by the plasma oxidation. Additional analyses such as transmission electron microscopy should be conducted to clarify the detailed structure of AuO_x_ layer on Au.

The suppression of *V*_Dirac_ shifts between dark and illuminated conditions presented in Fig. 2b implies that the formation of trapping sites on SiO_2_ is well eliminated by the natural oxidation process. Even with reduction of the *V*_Dirac_ shift, photosensitivity appears at *V*_GS_ > *V*_Dirac_. Figure 2c shows that the magnitude of photoresponse at *V*_GS_ > *V*_Dirac_ increases concomitantly with increase of the sweep rate. No significant photoresponse was observed (Fig. S5) in the case of 4-wire measurements, which indicates that the photoinduced modification of contact resistance serves an important role in the development of the photoresponse. Figure S6a and S6b respectively show transfer characteristics of G-FET (another device) measured at various wavelengths and *V*_DS_. No significant wavelength dependence is apparent in the visible region. Additionally, photoresponse Δ*I*_DS_ is proportional to *V*_DS_, with no significant nonlinear dependence for *V*_DS_ of 10–100 mV. Consequently, the observed photoresponses are not modified by the *V*_DS_, but are governed by the contact resistance at the graphene–electrode interface under the measurement conditions.

To clarify the photoinduced modulation of contact resistance, the transient photoresponse immediately after the light illumination was investigated. As portrayed in Fig. 3a, we applied *V*_GS_ pulse of 0 V (<*V*_Dirac_) for 2 ms, which corresponds to the gate bias for hole doping. Subsequently, the light pulse (light emitting diode (LED) with center wavelength of 623 nm, see Fig. S7 for more detail including experimental setup) was exposed at 4 ms after the application of *V*_GS_ = 50 V (>*V*_Dirac_), where *V*_DS_ = 100 mV. Figure 3a shows that *I*_DS_ responds quickly depending on the *V*_GS_ pulse within the time constant for our measurement setup (cutoff frequency: ~100 kHz). The noise floor of the measurement setup for the transient current is ~1 nA/Hz^1/2^, which is determined mainly by a high voltage amplifier for gate switching. The photocurrent still flows continuously after the light exposure is halted. This phenomenon is applicable to the optical memory, where the *V*_GS_ pulse of 0 V acts as the reset function. Furthermore, repeated application of this time sequence with 100 Hz provides a stable photoresponse with no remarkable degradation in vacuum.

Figure 3b shows the transient photocurrent Δ*I*_DS_ immediately after light exposure with various light intensities of 2.9–24 pW (29–240 μW/cm^2^) determined by the channel size (5 × 2 μm) of G-FET, where Δ*I*_DS_ was defined as the difference between the transient photoresponse of *I*_DS_ and the dark transient current immediately before the light exposure. As shown in the figure, a two-step increase of the photocurrent is apparent. The width of the plateau for the first step decreases concomitantly with increasing light intensity. For the second step, the photocurrent increases gradually with a certain time constant after light exposure. It saturates to certain values depending on the light intensity. With light exposure higher than 8 pW, the saturated Δ*I*_DS_ becomes almost equal to 2.5 μA.

Figure 3c portrays the pulse width dependence of the transient response. The value of Δ*I*_DS_ at the plateau for the second step increases concomitantly with increase of the light pulse width and saturates. Results also show that the time constant for the increase of Δ*I*_DS_ at the second step is independent of the light pulse width. Figure 3d shows the total number of irradiated photons and *N*_ph_ dependence of the Δ*I*_DS_ at plateau for the second step. It is readily apparent that the Δ*I*_DS_ at plateau for the second step is controlled by *N*_ph_ with the relation of Δ*I*_DS_ ∝ *N*_ph_^2^ at *N*_ph_ < ~10^5^. Further increase of *N*_ph_ induces the saturation of Δ*I*_DS_. It is noteworthy that the maximum photoresponse defined by the ratio of Δ*I*_DS_ and light intensity reaches ~3.1 × 10^5^ A/W (noise equivalent power (NEP): ~3 × 10^−15^ W/Hz^1/2^) with the optical memory function. As shown in an inset of Fig. 3d, the Δ*I*_DS_ for the first step also depends on the number of irradiated photons. From the perspective of a charge accumulation type device such as a charge-coupled device for single-photon detection, we estimate the maximum photosensitivities for single-photon sensing to be ~60 pA/photon for the first step and ~25 pA/photon for the second step at room temperature. We believe that single-photon level detection at room temperature can be realized by the G-FET with the Au oxide on the electrodes using a commercially available low-noise and high-speed amplifier because of the high photosensitivity of NEP ~3 × 10^−15^ W/Hz^1/2^, which is comparable to the commercially available photomultiplier tube.

To elucidate the retention property of the memory function of the photosensitivity, transient photoresponses on the order of seconds were investigated. Figure 4a shows that we first applied *V*_GS_ with a step function of 0–50 V through *V*_Dirac_, which corresponds to the gate bias for hole doping to electron doping. Subsequently, the light was irradiated for 2 s. The bottom of Fig. 4a shows a stepwise increase of photocurrent initiated by the illumination. It is noteworthy that the photocurrent still flows continuously with a gradual decrease even after the removal of the illumination, which closely resembles “persistent photocurrent”, which is the memory action of the photocurrent. In contrast, the dark current increases gradually with time and coincides finally with the photocurrent at ~14 s. Figure 4b shows that the retention property of Δ*I*_DS_ exhibits a single exponential decay with a retention time constant τ of ~5.2 s, which is sufficient for the conventional image sensor application at room temperature. This temporal variation of Δ*I*_DS_ results in the sweep rate dependences observed in Figs 1 and 2. Figure 4c shows that the activation energy of τ estimated from an Arrhenius plot is quite small, ~18 meV, which is much less than that for the thermal energy at room temperature. This low temperature dependence implies the presence of a small barrier or shallow trap of the Au oxide at the interface to the dark currents.

Reportedly, graphene-MoS_2_ hybrid structures show gate-tunable persistent photoconductivity with a retention time longer than hours even at room temperature, which is induced by the trapped charge at MoS_2_ layer adjacent to the graphene channel^15^. In this case, the energy depth of the trapping site is ~0.8 eV, which is much deeper than that of our device, which results in the longer retention time for persistent states. The combination of organic photosensitive material of poly(3-hexylthiophene) (P3HT) has also reported for the generation of photogenerated charge, where a piezoelectric Pb(Zr_0.2_Ti_0.8_)O_3_ (PZT) substrate was used to facilitate the accumulation of photogenerated charge by polarization of PZT^16^. The time constant for the photoresponse is on the order of seconds. One can expect that the polarization of PZT would be changed by the gate voltage similar to the G-FET with ferroelectric gate insulator^30^,^31^,^32^, so that this slow response might be applicable to the memory function. These photoresponses are induced by the gating effect of the trapped charge adjacent to the graphene channel, different from our proposed device. Consequently, the time constant for photoresponses immediately after the irradiation of light is on the order of seconds, which is much longer than our device.

Based on experimentally obtained results, we consider that the contact resistance modification stimulated by the light irradiation derives mainly from the relaxation of the trapped charge at the Au oxide as follows. First, we consider the band diagram of our device. The work function of gold covered with an atomically thin AuO_x_ layer is ~5.5 eV, which is larger than that of the bulk gold (~5.1 eV)^33^. The bulk Au_2_O_3_, which is the most stable state of the Au oxide, is the semiconductor with the bandgap of 0.83 eV, whereas Au_2_O, which has lower valency, is metallic (no bandgap)^34^. Based on the XPS spectra, as discussed in Fig. 2a, the Au oxide at the interface is expected to be a narrower bandgap semiconductor or metallic layer. The work function of graphene is ~4.6 eV^35^. The resultant schematic potential profile based on the band diagram (Fig. 5a) at *V*_GS_ = *V*_Dirac_ is presented in Fig. 5b. It is noteworthy that electron–hole asymmetric transfer characteristics attributed to the charge transfer at the metal/graphene have been reported^10^,^17^. In this case, the graphene near the contact is expected to behave as p-type because of the donation of electrons from graphene to Au through the atomically thin AuO_x_ layer. Assuming that the AuO_x_ layer acts as the electron trapping site, a part of AuO_x_ layer would be negatively charged. The assumption might be made that the hydrated form of Au_2_O_3_ is weakly acidic^36^. Therefore, we can expect that the AuO_x_ tends to accept the negative charge.

At *V*_GS_ < *V*_Dirac_ corresponding to the hole-doping regime, the entire graphene region between the source and drain electrodes is p-type (p-p-p junction), providing the lower contact resistance at translation region indicated in Fig. 5c. Further electron donation from the graphene to the Au oxide (hole injection from Au to graphene through the AuO_x_ layer) occurs at *V*_GS_ < *V*_Dirac_, so that the excess electrons would be trapped at the AuO_x_ layer.

Subsequent change of *V*_GS_ < *V*_Dirac_ to *V*_GS_ > *V*_Dirac_ induces electron doping on the graphene channel (n-type at channel), whereas the graphene near the contact remains p-type because of the work function difference, as presented in Fig. 5c. Consequently, the p-n-p type junction is formed through the channel. The formation of a p-n junction near the contact at *V*_GS_ > *V*_Dirac_ causes a higher contact resistance^10^. Based on this model, the trapped electron at the Au oxide on the electrode interfaces can be expected to contribute to the modulation of the contact resistance as follows. The excess trapped electrons induce the excess holes at the interface and screens; alternatively, they terminate the electric field from the back gate as presented schematically in Fig. 5d. At the translation region from p-type to n-type, the excess trapped electrons cause a wider p-type region, because of the pinned work function difference and lower electron concentration at graphene.

Under light illumination, the trapped electrons at the Au oxide are relaxed quickly, leading to lower contact resistance than that in a dark condition. Reportedly, the efficient charge transfer is induced by light irradiation at the interface between the graphene and AuO_x_ layer because of the strong electric field between them^26^. In our case, the additional electric field (p-n junction) at the translation region enhances the charge separation of photo-generated hole-electron pair, as presented in Fig. 5d. The generated holes drifted toward electrode would compensate the trapped excess electrons. Additionally, direct photoexcitation of trapped excess electrons near the channel can be expected because of the narrow-gap semiconducting characteristics of the AuO_x_ layer, which might also decrease the work function difference between the graphene and Au electrodes. The electrons trapped distant from the electrode edge give no contribution to the contact resistance modification. This is because the back gate induced electric field is fully screened by the bulk Au layer underneath the atomically thin AuO_x_ layer, which results in no gate polarity dependence. Therefore, we can expect that the active region for the photosensitivity is the translation region at the p-n junction near the electrodes. Additional experiments using methods such as scanning photocurrent microscopy are expected to be necessary to clarify the mechanism.

After removal of the light at *V*_GS_ > *V*_Dirac_, the trapping sites for the excess electrons are still empty states, so that the photo-modulated translation region remains in the light irradiation states. Therefore, the optical memory function is visible in our device. No hole exists in the channel region because of the n-type graphene channel. Therefore, the residual trapped excess electrons are expected to be released gradually from the AuO_x_ layer toward the thermal equilibrium state. Consequently, the gradual increase of *I*_DS_ under the dark condition observed in this experiment is likely to be the result of the decrease of the contact resistance induced by the trapped charge relaxation. The retention time for the optical memory operation is determined by the release rate of the trapped excess electrons at AuO_x_ layer. Finally, the full relaxation of trapped excess electron (thermal equilibrium state at *V*_GS_ > *V*_Dirac_) is expected to induce the narrower translation region as shown in Fig. 5f, which gives lower contact resistance. The repeated cycle from Fig. 5c–e results in the optical memory operation of the G-FET with the AuO_x_ layer.

The two-step process presented in Fig. 3 suggests the presence of relaxation processes of two kinds for trapping sites at the Au oxide. The transient response of Δ*I*_DS_ of the first step shows linear dependence of *N*_ph_ while *N*_ph_^2^ for the second step. The order difference of *N*_ph_ dependences of photoresponses implies the presence of two mechanisms for the photosensitivity observed in this study as discussed above. Although a direct thermal effect induced by the light absorption might induce the thermal excitation of trapped charge, the temperature rise induced by the light irradiation (30 μW/cm^2^ for 3 pW) is negligibly small. Whereas the photo-excited excess carrier at the graphene channel except for the p-n region should also be regarded as this relaxation mechanism, this effect is also negligible because of the fast relaxation time of the photo-excited charge^6^,^7^ in graphene. Direct contribution of the photothermoelectric effect on the observed photoresponse may also be negligible because the light intensity is in the order of pW which is quite small for sufficient photothermal effect and no memory effect should be observed. However, the Fermi levels near source and drain electrode are different because of the charge accumulation at the interface traps, which results in the different photothermoelectric voltages at the electrodes. Thus, the photothermoelectric effect is one of candidates for the origin of the relaxation of the accumulated charge at the trap. Consequently, the possible mechanism for the observed photosensitivity is the contact resistance modification induced not only by the direct excitation of trapped electrons at AuO_x_ but also the photo-excited excess carrier at the p-n region of graphene or photothermoelectric effect near the electrode. The photocurrents for the first and second step were saturated respectively by irradiation of the limited number of photons of ~5 × 10^3^ and ~1 × 10^5^ photons/(2 × 5 μm^2^), as portrayed in Fig. 3d. It can be expected that the number of photons for the saturation is related to the density of traps that contribute to the photoresponse. Assuming the translation region to be 0.2 μm^37^ for each contact, the densities of traps for photoresponse for first and second steps are estimated as roughly 10^14^ and 10^18^ cm^−3^, respectively, where the number of photons absorbed in the translation region of graphene is estimated from the light absorption coefficient of graphene of 2.3%. Further study must be undertaken to ascertain the detailed mechanisms of the photoresponse observed here.

In summary, we fabricated a highly photosensitive G-FET (NEP of ~3 × 10^−15^ W/Hz^1/2^) with optical memory function with a retention time of ~5 s at room temperature without a complicated process. It acts as the functional device for photoinduced charge sensing. High photosensitivity was realized by modification of effective contact resistance because of the light-assisted relaxation of the trapped charge at the AuO_x_ layer. Relaxation processes of two kinds were identified with a direct excitation of trapped charge at AuO_x_ layer and/or the photo-excited excess carrier at the p-n region of graphene near the electrode. These findings are expected to open the way to realization of graphene photodetectors with high sensitivity up to the single-photon detection level.

## Additional Information

**How to cite this article**: Ishida, S. *et al.* Highly photosensitive graphene field-effect transistor with optical memory function. *Sci. Rep.* **5**, 15491; doi: 10.1038/srep15491 (2015).

## Supplementary Material

Supplementary Information

## Figures and Tables

**Figure 1:**
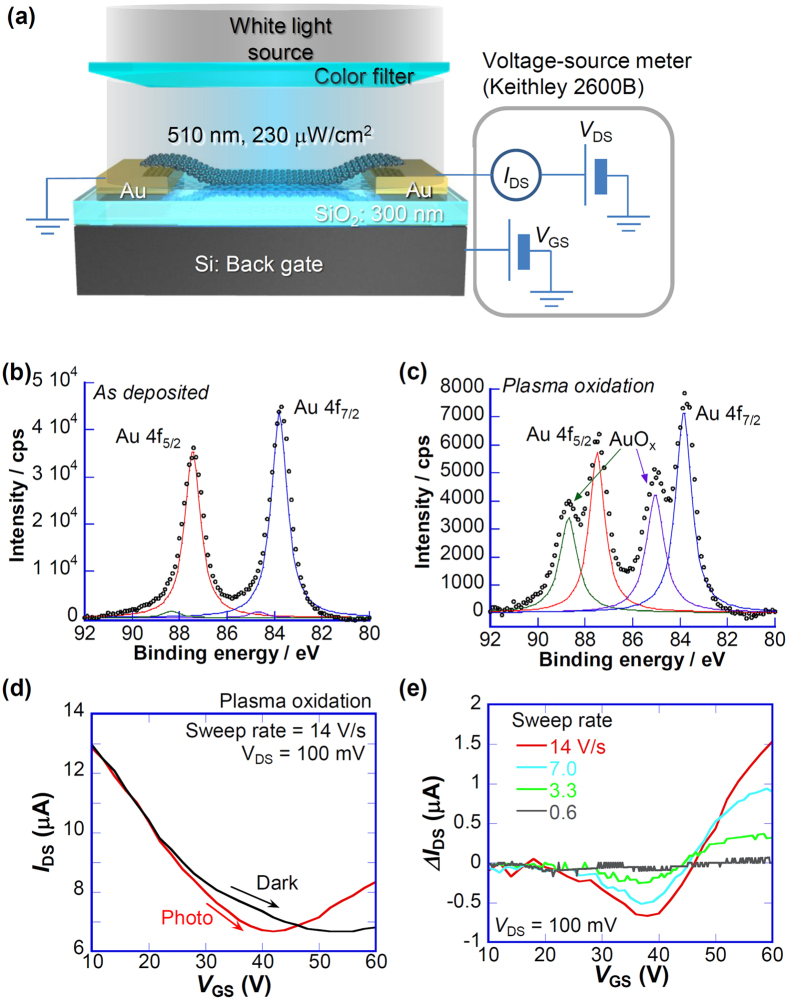
Transfer characteristics of G-FET with Au oxide formed by plasma oxidation. (**a**) Schematic illustration of G-FET with Au oxide on source and drain electrodes. (**b**,**c**) respectively portray XPS spectra of Au 4f peaks before and after plasma oxidation. (**d**) Transfer characteristics of G-FET with Au oxide formed by plasma oxidation measured under conditions of dark and light illumination. (**e**) *V*_GS_ dependence of Δ*I*_DS_ with various sweep rates of *V*_GS_.

**Figure 2:**
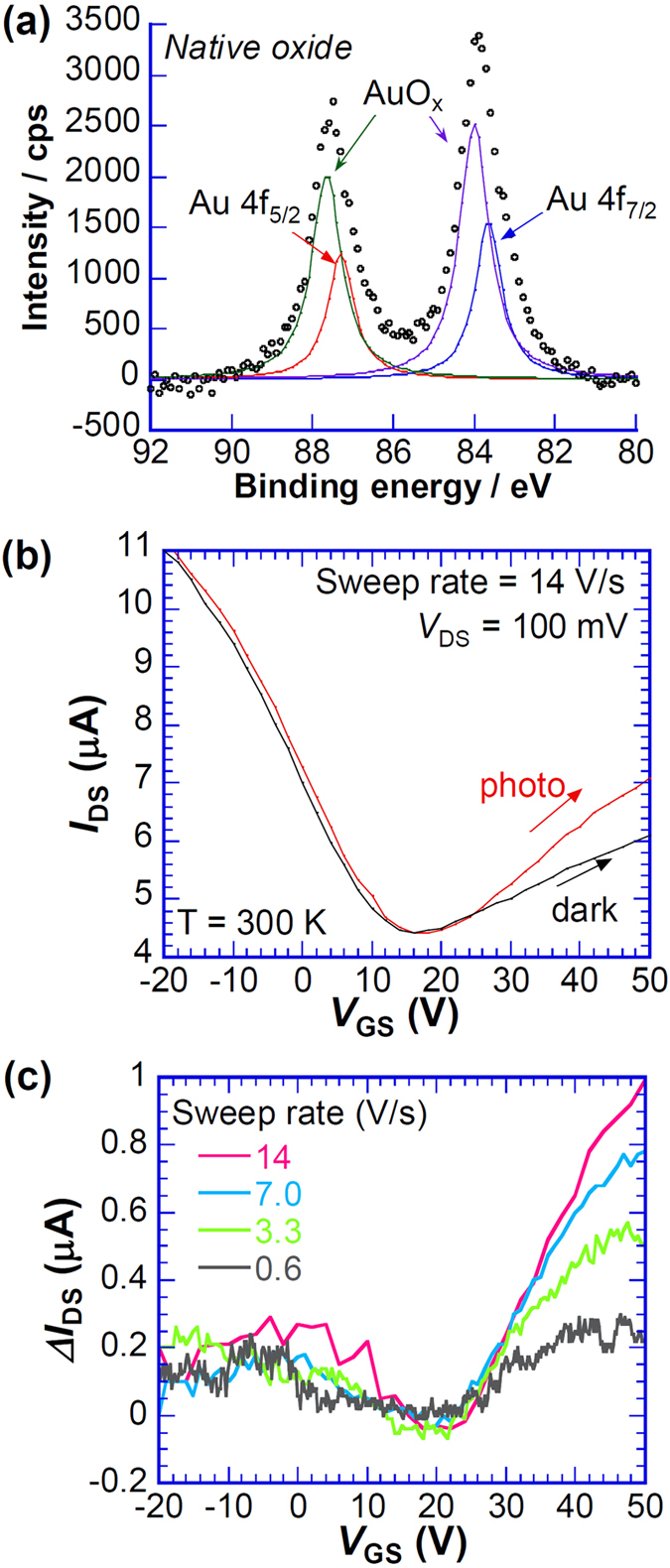
Transfer characteristics of G-FET with native Au oxide. (**a**) XPS spectrum of Au 4f peaks after natural oxidation. (**b**) Transfer characteristics of G-FET with Au oxide formed by natural oxidation measured under conditions of dark and light illumination. (**c**) *V*_GS_ dependence of Δ*I*_DS_ with various sweep rates of *V*_GS_.

**Figure 3:**
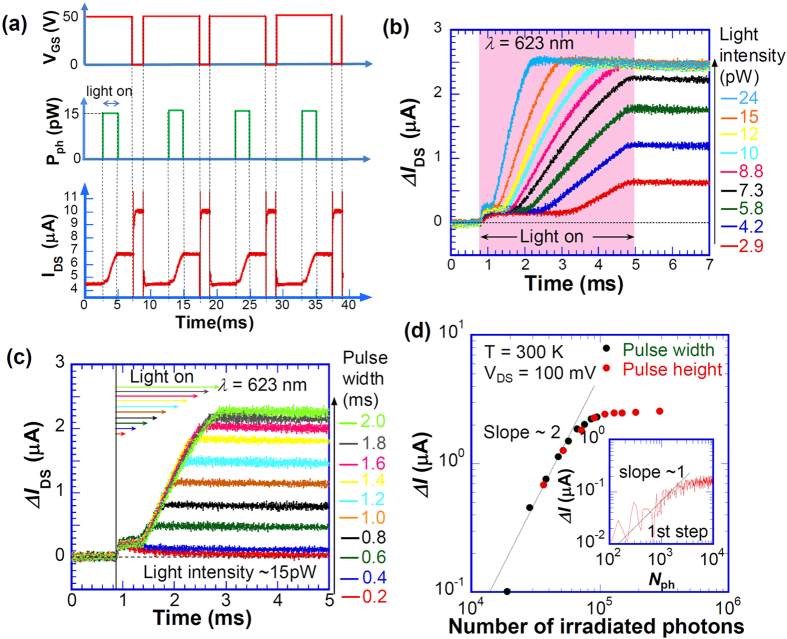
Transient responses of Δ*I*_DS_. (**a**) Schematic timing chart of *V*_GS_ and light irradiation. The bottom panel shows repeated transient responses for *I*_DS_. (**b**) Temporal variation of Δ*I*_DS_ with various light intensities. (**c**) Temporal variation of Δ*I*_DS_ with various light pulse-widths. Arrows indicate the pulse widths. (**d**) Number of irradiated photons dependence of the photoresponse for the second step. Inset shows the number of irradiated photons dependence of the photoresponse for the first step.

**Figure 4:**
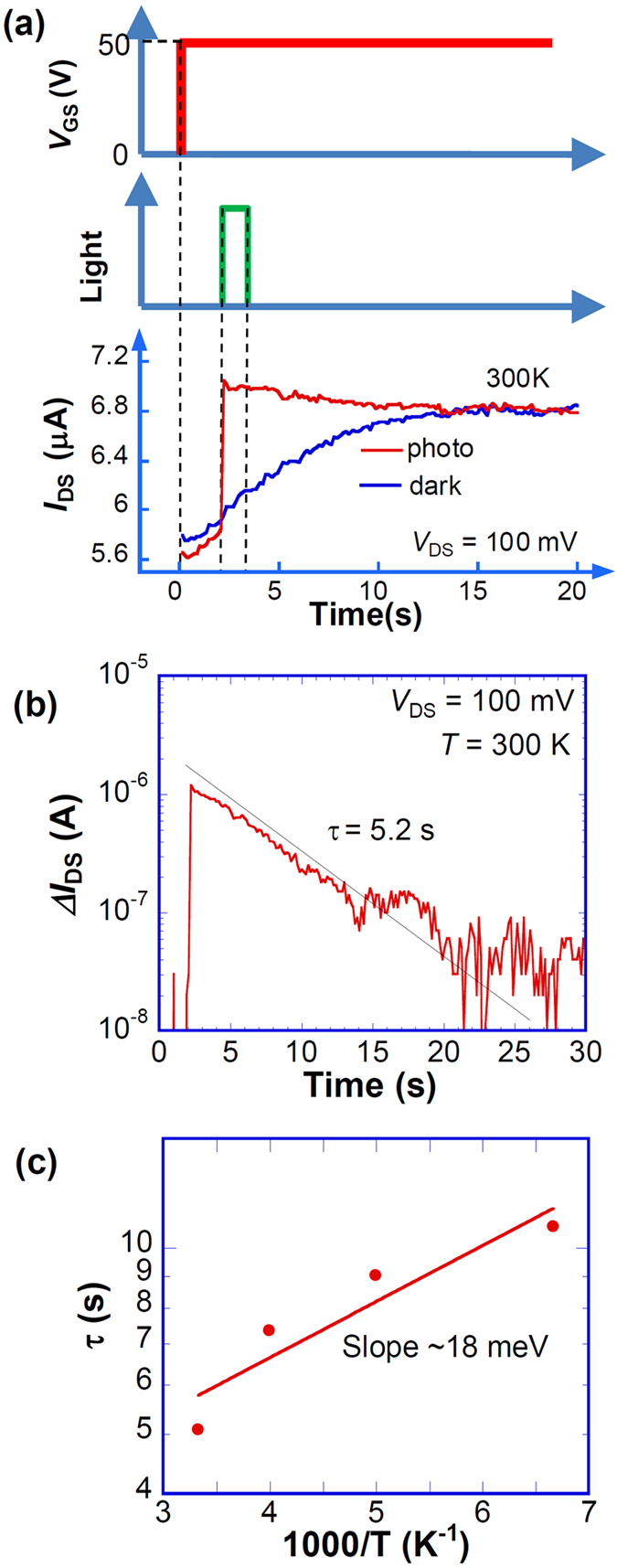
Retention property of photoinduced *I*_DS_. (**a**) Schematic timing chart of *V*_GS_ and light irradiation. The bottom panel shows retention property induced by relaxations of dark and photocurrents. (**b**) Semi-log plot of retention property of Δ*I*_DS_. (**c**) Arrhenius plot of the retention time constant obtained from the temporal variation of Δ*I*_DS_, where a solid line is a guide for eyes.

**Figure 5:**
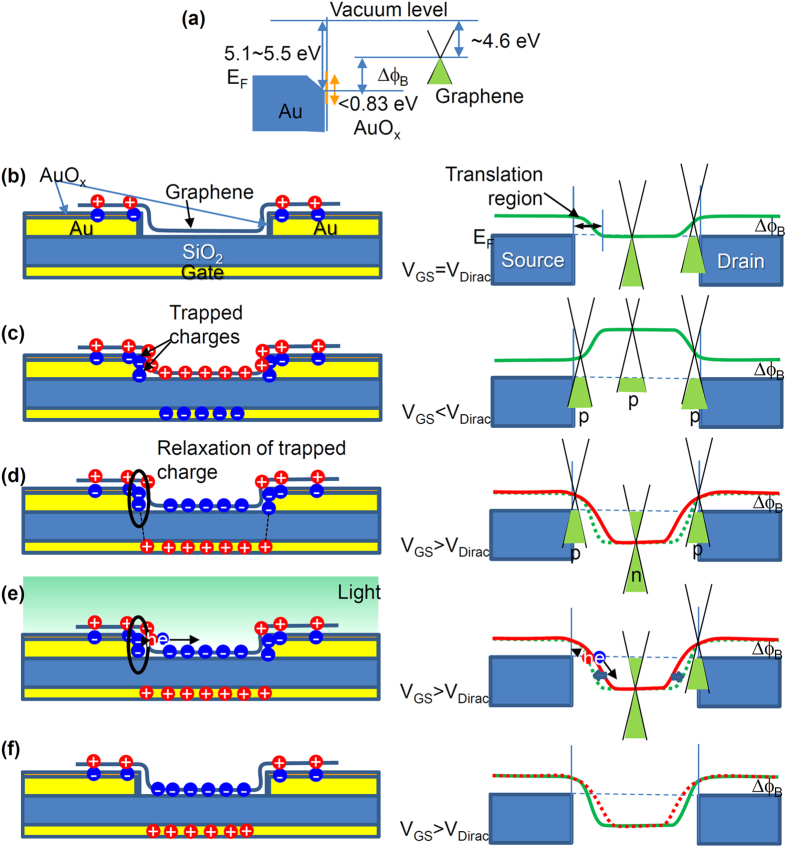
Schematic potential profile. (**a**) Schematic band diagram of Au/atomically thin AuO_x_/Graphene contact. (**b**–**d**) Schematic charge distributions and potential profiles of *V*_GS_ = *V*_Dirac_, *V*_GS_ < *V*_Dirac_, and *V*_GS_ > *V*_Dirac_ with light irradiation, respectively. The excess trapped electrons are denoted by a circle in (**d**). Red solid and green dotted lines in (**d**) of the right respectively show immediately after *V*_GS_ changed to *V*_GS_ > *V*_Dirac_ and for equilibrium state. (**e**) Schematic charge distribution and potential profile after light illumination and (**f**) fully relaxed states at *V*_GS_ > *V*_Dirac_.
