# Supplementary material for: Highly photosensitive graphene field-effect transistor with optical memory function
Source: Sci Rep. 2015 Oct 20;5:15491. doi: 10.1038/srep15491 (PMC4611878; doi:10.1038/srep15491)
Supplement: Supplementary Information [file srep15491-s1.pdf]

Supplementary information

Highly photosensitive graphene field-effect transistor with optical memory function

Shohei Ishida[1], Yuki Anno[1], Masato Takeuchi[2], Masaya Matsuoka[2], Kuniharu Takei[1], Takayuki Arie[1] and Seiji Akita[1],[*]

[1]Department of Physics and Electronics, Osaka Prefecture University

[2]Department of Applied Chemistry, Osaka Prefecture University

1-1 Gakuen-cho, Naka-ku, Sakai, Osaka 599-8531, Japan

1. Raman spectroscopy of grown graphene

To clarify the quality of graphene used in this experiment, we have performed Raman spectroscopy. Figure S1 shows a typical Raman spectrum of our graphene. The intensity ratio of 2D/G bands is larger than 2, where G and 2D bands are peaks observed at 1590 and 2690 cm^{-1}, respectively.[1] This indicates that the graphene is monolayer. Additionally, very small D band peak is appeared resulting in the presence of small amount of defects.

2. Photoresponse of G-FET without oxidation of Au-electrodes

Figure S2a represents the transfer characteristics of G-FET without oxidation Au-electrodes under gate voltage (V_{GS}) sweep rate of 7 V/s with or without light illumination (510nm, 230 μW/cm^2) measured at vacuum condition (~10^{-3} Pa), where the transfer characteristics were measured using a conventional 2 channel voltage-source meter (Keithley 2600B). The difference between transfer characteristics of dark and illuminated conditions are quite small in comparison of the G-FET with oxidized electrodes shown in Figure 1d in main text. The

[*] E-mail: akita@pe.osakafu-u.ac.jp

transient photoresponse (623 nm, 23 pW for 2×5 μm channel) of drain current (I_{DS}) is also shown in Figure S2b. Even in the transient measurement, the photoresponse is hardly observed. Thus, the oxidation process is essential for the development of photoresponse.

3. Sweep rate dependence of transfer characteristics of G-FET

Figure S3a and S3b show transfer characteristics of G-FET with oxidized electrodes measured under the light illuminated and dark conditions, respectively. The transfer characteristics for light illumination with different sweep rates are almost identical. However, the transfer characteristics for dark condition shows strong sweep rate dependence at $V_{GS} > 20$ V, while the transfer characteristics at $V_{GS} < 20$ V corresponding to the hole doping region are almost identical. As shown in Figure 2d and 2e in main text, the photoresponse observed in this study mainly comes from the relaxation process of accumulated charge enhanced by the light illumination as described in main text.

To make clear the hysteresis on the transfer characteristics, the transfer characteristics for all sweep rates with or without light illumination are shown in Figure S4. The hysteresis observed under light illumination increased with decreasing the sweep rate, which originates from the charge accumulation at the gate insulator as usually observed[2]. However, under the dark condition, anomalous behavior is observed at $V_{GS} > 20$ V at higher sweep rate (7 or 14 V/s). When the gate voltage decreases, the charge neutral point (V_{Dirac}) shifts to the value for the light illumination and I_{DS} around V_{Dirac} follows the trace measured with light illumination. This implies that the accumulated charge at $V_{GS} < V_{Dirac}$ is released at $V_{GS} > V_{Dirac}$.

4. Four-terminal measurement

In order to clarify the effect of contacts with Au oxide, we performed the 4-wire measurement. Figures S5a and S5b show the transfer characteristics of G-FET with Au-oxides formed by oxygen plasma oxidation measured by 2-wire and 4-wire measurements, respectively. The photoresponse even for the 4-wire measurement is observable. The results indicates that the photoresponse (photo-relaxation of accumulated charge) is occurred not only the contacts but also the graphene channel.

On the other hand, as shown in Figures S5c and S5d, while the photoresponse of G-FET with native oxide is disappeared for 4-wire measurement, the photoresponse can be clearly observed in the case of 2-wire measurement. This indicates that the contribution of graphene channel to the photoresponse is much smaller than that of contacts. Consequently, this result strongly supports our hypothesis that the accumulated charge at Au-oxide is relaxed by the photoinduced carrier near the contact.

5. Wavelength and V_{DS} dependences

Figure S6a shows the photoresponse measured under various wavelengths with a constant light intensity of 12 μW/cm^2 for each wavelength, where the wavelength was varied from 410 to 770 nm using respective color filters. While the measured device is different from the device described in the main text, all of the devices measured show similar wavelength dependences. This implies that the optical memory effect works well for all of the wavelengths measured here. Note that the photoresponse ΔI_{DS} is saturated at relatively short time constant less than a few ms even under the weak light intensity as discussed in the transient states shown in Fig. 3b and 3c. As a result, the photoresponse obtained here shows no significant wavelength dependence, where the sweep rate of the gate voltage is limited to be 14 V/s, so that the required time for the gate sweep was ~ 5 s for V_{GS} from -20 to 50 V.

To clarify the I_{DS}-V_{DS} dependence on the output characteristics, the photoresponse ΔI_{DS} obtained from the transfer characteristics with V_{DS} of 10 - 100 mV were investigated as shown in Fig. S6b-S6d. The magnitude of photoresponse shows linear dependence of the V_{DS}. This linear dependence indicates that the photoresponse observed here is not modified by the V_{DS} but governed by the contact resistance at the graphene-electrode interface under the measurement conditions.

6. Experimental setup for transient response

Figure S7a shows Schematic circuit for the transient measurement, where the LED with a center wavelength of 623 nm (Toshiba TLSH180P), which emission spectrum is shown in Fig. S7b, was used as the pulse light source, where the light beam diameter is larger than 10 mm on the sample surface. The fast swing of the V_{GS} was realized by using a combination of a function generator (Tektronix AFG3252) and the high-voltage amplifier. The transient current was recorded by a digital oscilloscope through the current-voltage amplifier (NF LI-76).

7. Reproducibility of device

Figure S8 shows a data set taken from other device, which shows the higher photosensitivity than that shown in the main text. The higher sensitivity may be induced by the Au oxides because of the natural oxidation process.

References

1 Ferrari, A. C. *et al.* Raman Spectrum of Graphene and Graphene Layers. *Phys. Rev. Lett.* **97**, 187401, (2006).

2 Wang, H., Wu, Y., Cong, C., Shang, J. & Yu, T. Hysteresis of Electronic Transport in Graphene Transistors. *Acs Nano* **4**, 7221-7228, (2010).

Figure captions

Figure S1 Raman spectrum of graphene. A typical Raman spectrum of our graphene, where G and 2D bands are peaks observed at 1590 and 2690 cm^{-1}, respectively. No base line correction was performed.

Figure S2 Photoresponse of G-FET without oxidation of Au-electrodes. a, Transfer characteristics of G-FET without Au-oxidation process. **b**, Transient photoresponse of G-FET without oxidation process of electrodes. Top and middle panels show the timing chart for V_{GS} (from 20 to 100 V) and light irradiation (dark to 23 pW, 610 nm), respectively. Bottom panel represents the transient response of I_{DS}.

Figure S3 Sweep rate dependence of transfer characteristics. a and b, Transfer characteristics under, **a,** light illumination (510nm, 230 μW/cm^2) and, **b,** dark condition measured in vacuum (~10^{-3} Pa) with various sweep rate of V_{GS}.

Figure S4 Hysteresis of transfer characteristics. a and b, Transfer characteristics for the V_{GS} sweep rate of 14 V/s measured under, **a**, light illumination and, **b**, dark. **c and d,** Transfer characteristics for the V_{GS} sweep rate of 7 V/s measured under, **c**, light illumination and, **d**, dark. **e and f,** Transfer characteristics for the V_{GS} sweep rate of 3.3 V/s measured under, **e**, light illumination and, **f**, dark. **g and h,** Transfer characteristics for the V_{GS} sweep rate of 0.6 V/s measured under, **g**, light illumination and, **h**, dark.

Figure S5 Two-wire and 4-wire measurement. a and **b** show gate voltage dependence of channel conductance of G-FET with plasma oxidized Au-electrodes measured by 2-wire and 4-wire measurements, respectively. **c** and **d** represent gate voltage dependence of channel conductance of G-FET with native oxide on Au-electrodes measured by 2-wire and 4-wire measurements, respectively. The bias current for the 4-wire measurement was set to be 1 μA.

Figure S6 Wavelength and V_{DS} dependences of photoresponses. a Photoresponse measured under the various wavelength from 410 to 770 nm with a constant light intensity of 12 μW/cm^2, where the sweep rate of V_{GS} was 14 V/s for each wavelength. **b-d** photoresponse measured with V_{DS} = 10, 50, and 100 mV under the wavelength of 510 nm and the V_{GS} sweep rates of 3.3-14 V/s.

Figure S7 Experimental setup for the transient measurement. a Schematic circuit for the transient measurement. **b** Relative emission profile of the LED used as the pulse light source.

Figure S8 Reproducibility of the device. a and b Transfer characteristics and obtained photoresponse of G-FET with Au-oxidate. **c** Temperature dependence of retention time. **d-e** Transient photoresponses. **f** Number of irradiated photons dependence of the photoresponse for the second step. Inset shows the number of irradiated photons dependence of the photoresponse for the first step.

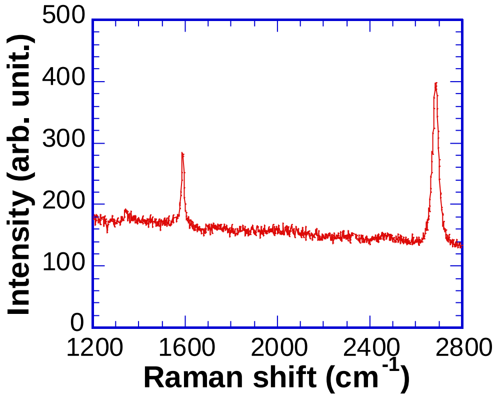

Intensity (arb. unit.)
500
400
300
200
100
0
1200
1600
2000
2400
2800
Raman shift (cm-1)

Figure S1

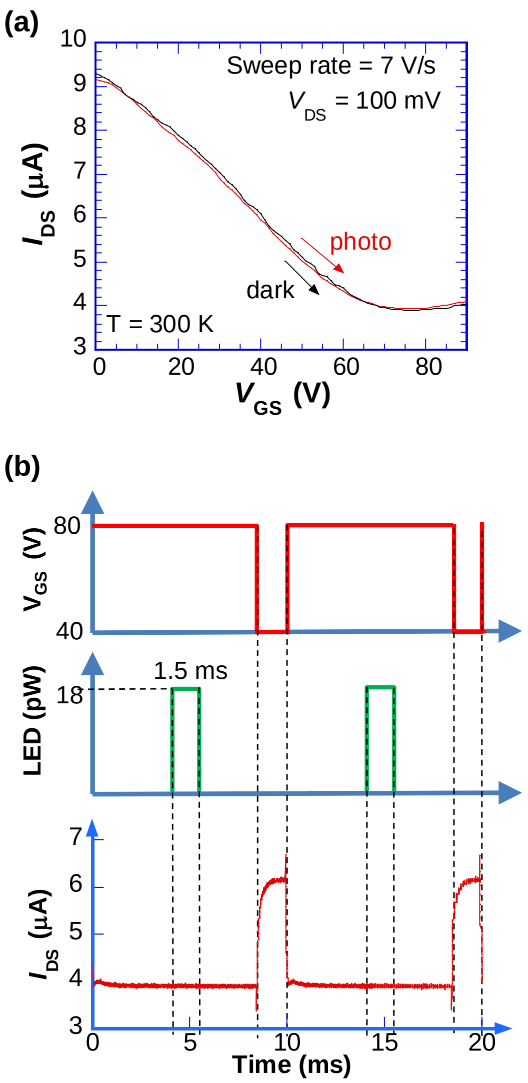

(a)
10
9
8
7
6
5
4
3
Sweep rate = 7 V/s
V DS = 100 mV
photo
dark
T = 300 K
0
20
40
60
80
I DS (μA)
V GS (V)
(b)
80
40
V GS (V)
1.5 ms
18
LED (pW)
7
6
5
4
3
I DS (μA)
0
5
10
15
20
Time (ms)

Figure S2

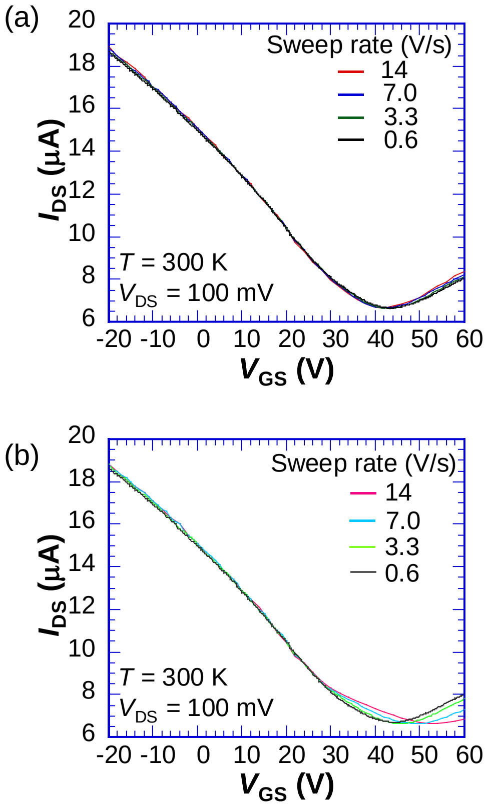

(a)
Sweep rate (V/s)
14
7.0
3.3
0.6
T = 300 K
V_DS = 100 mV
I_DS (μA)
V_GS (V)
(b)
Sweep rate (V/s)
14
7.0
3.3
0.6
T = 300 K
V_DS = 100 mV
I_DS (μA)
V_GS (V)

Figure S3

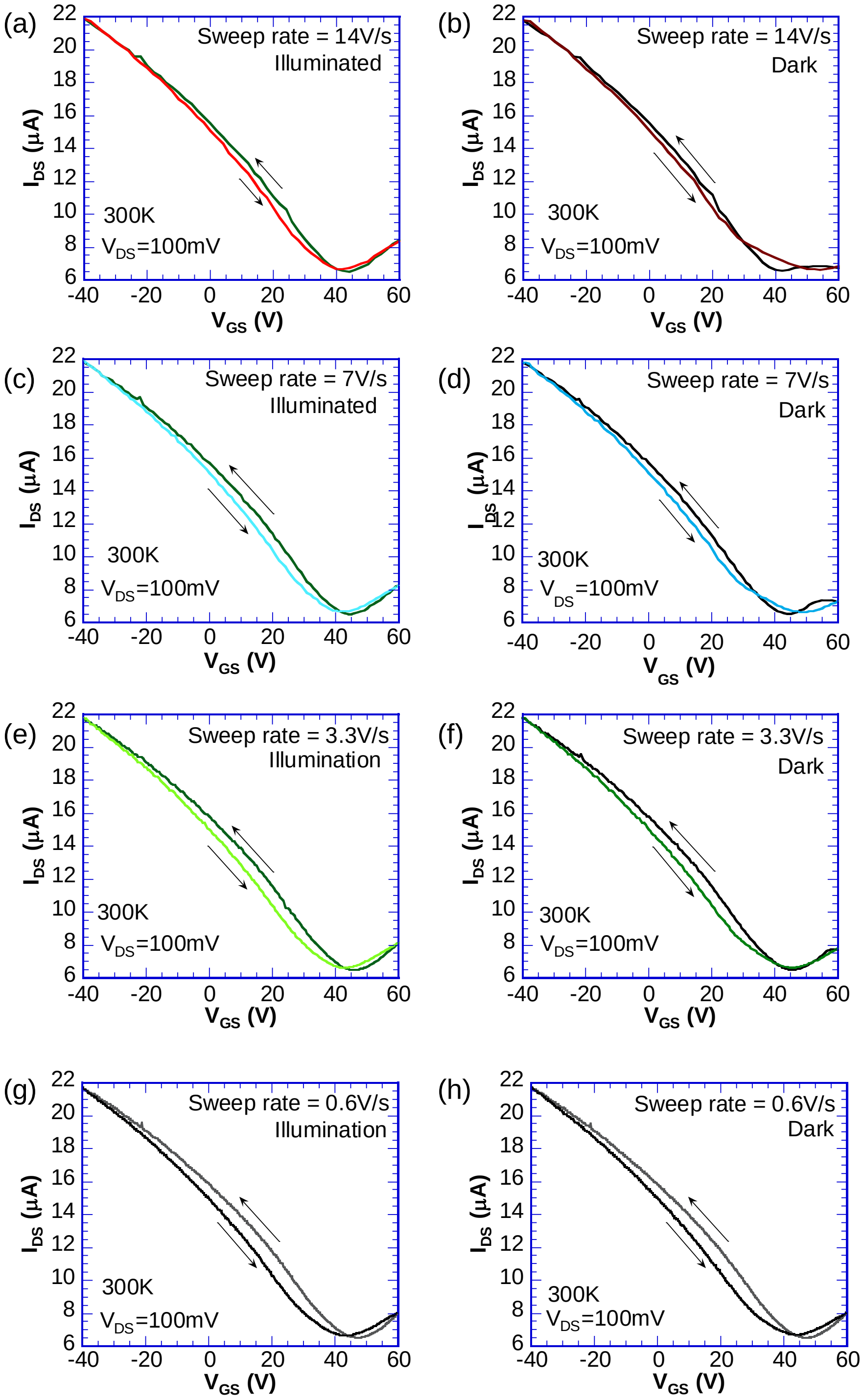
(a)
Sweep rate = 14V/s
Illuminated
300K
V_{DS}=100mV
(b)
Sweep rate = 14V/s
Dark
300K
V_{DS}=100mV
(c)
Sweep rate = 7V/s
Illuminated
300K
V_{DS}=100mV
(d)
Sweep rate = 7V/s
Dark
300K
V_{DS}=100mV
(e)
Sweep rate = 3.3V/s
Illumination
300K
V_{DS}=100mV
(f)
Sweep rate = 3.3V/s
Dark
300K
V_{DS}=100mV
(g)
Sweep rate = 0.6V/s
Illumination
300K
V_{DS}=100mV
(h)
Sweep rate = 0.6V/s
Dark
300K
V_{DS}=100mV
I_{DS} (μA)
V_{GS} (V)

Figure S4

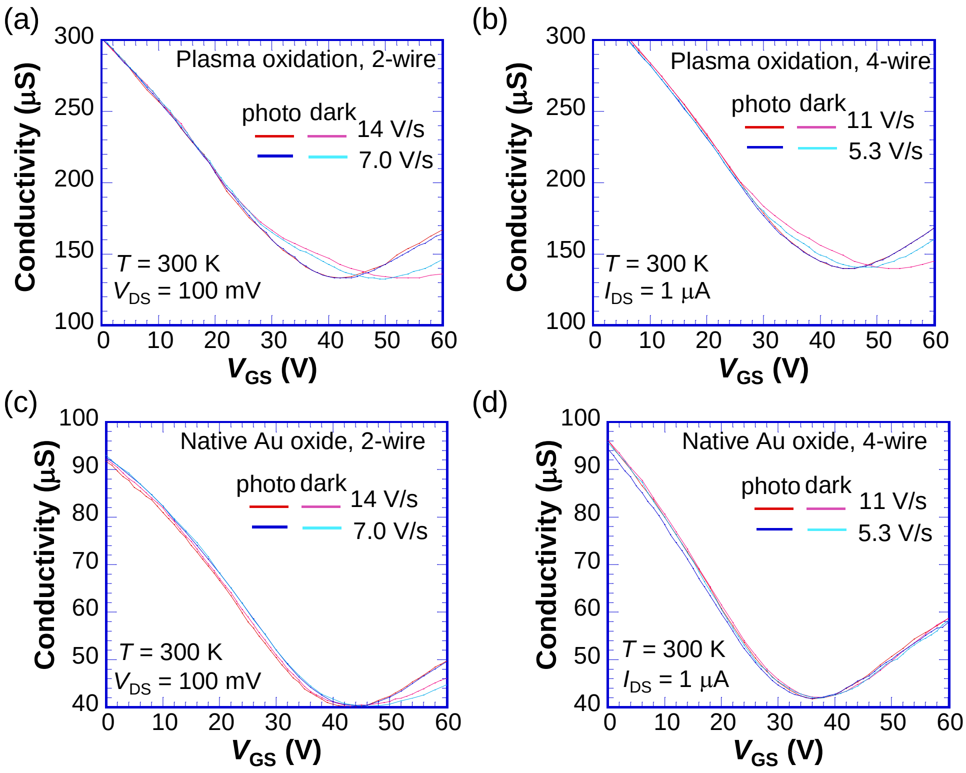
(a)
Plasma oxidation, 2-wire
photo dark
14 V/s
7.0 V/s
T = 300 K
V_DS = 100 mV
Conductivity (µS)
V_GS (V)
(b)
Plasma oxidation, 4-wire
photo dark
11 V/s
5.3 V/s
T = 300 K
I_DS = 1 µA
Conductivity (µS)
V_GS (V)
(c)
Native Au oxide, 2-wire
photo dark
14 V/s
7.0 V/s
T = 300 K
V_DS = 100 mV
Conductivity (µS)
V_GS (V)
(d)
Native Au oxide, 4-wire
photo dark
11 V/s
5.3 V/s
T = 300 K
I_DS = 1 µA
Conductivity (µS)
V_GS (V)

Figure S5

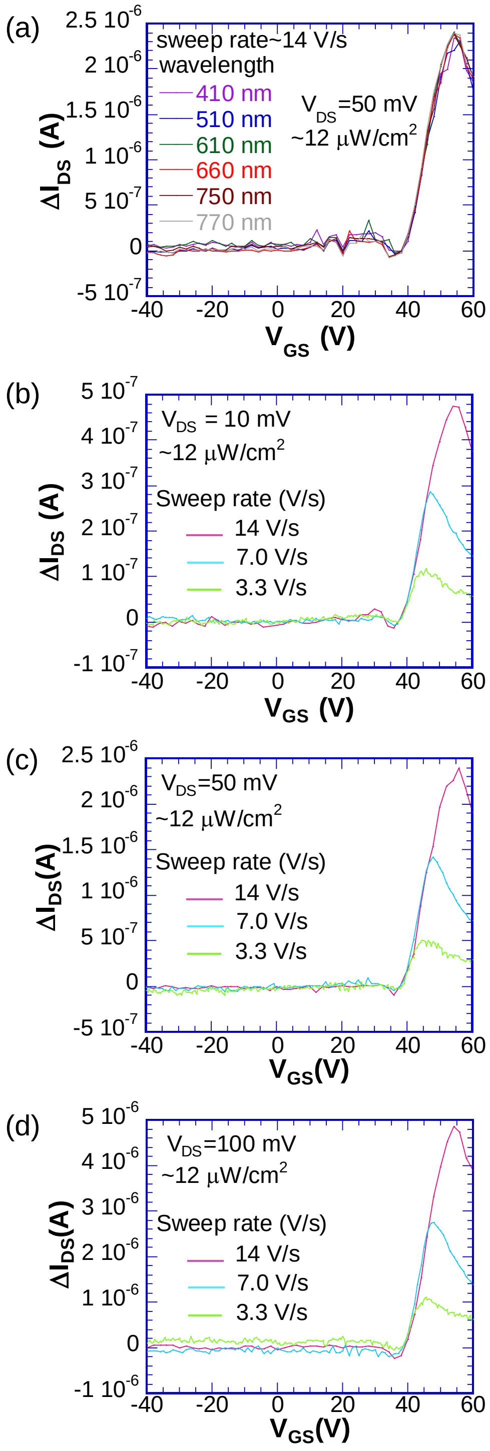
(a)
sweep rate~14 V/s
wavelength
410 nm
510 nm
610 nm
660 nm
750 nm
770 nm
V_DS=50 mV
~12 μW/cm^2
ΔI_DS (A)
V_GS (V)
(b)
V_DS = 10 mV
~12 μW/cm^2
Sweep rate (V/s)
14 V/s
7.0 V/s
3.3 V/s
ΔI_DS (A)
V_GS (V)
(c)
V_DS=50 mV
~12 μW/cm^2
Sweep rate (V/s)
14 V/s
7.0 V/s
3.3 V/s
ΔI_DS(A)
V_GS(V)
(d)
V_DS=100 mV
~12 μW/cm^2
Sweep rate (V/s)
14 V/s
7.0 V/s
3.3 V/s
ΔI_DS(A)
V_GS(V)

Figure S6

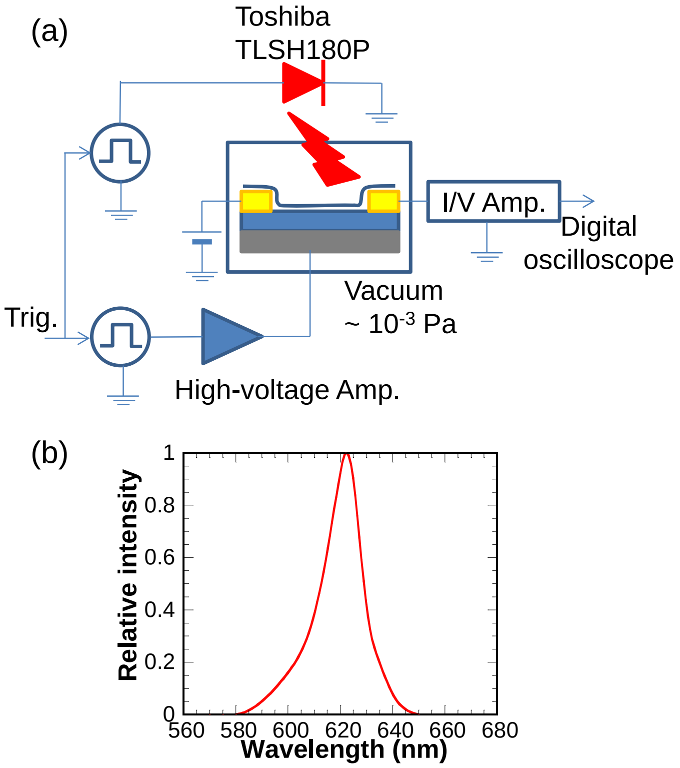
(a)
Toshiba
TLSH180P
I/V Amp.
Digital
oscilloscope
Vacuum
~ 10-3 Pa
Trig.
High-voltage Amp.
(b)
Relative intensity
Wavelength (nm)
1
0.8
0.6
0.4
0.2
0
560 580 600 620 640 660 680

Figure S7

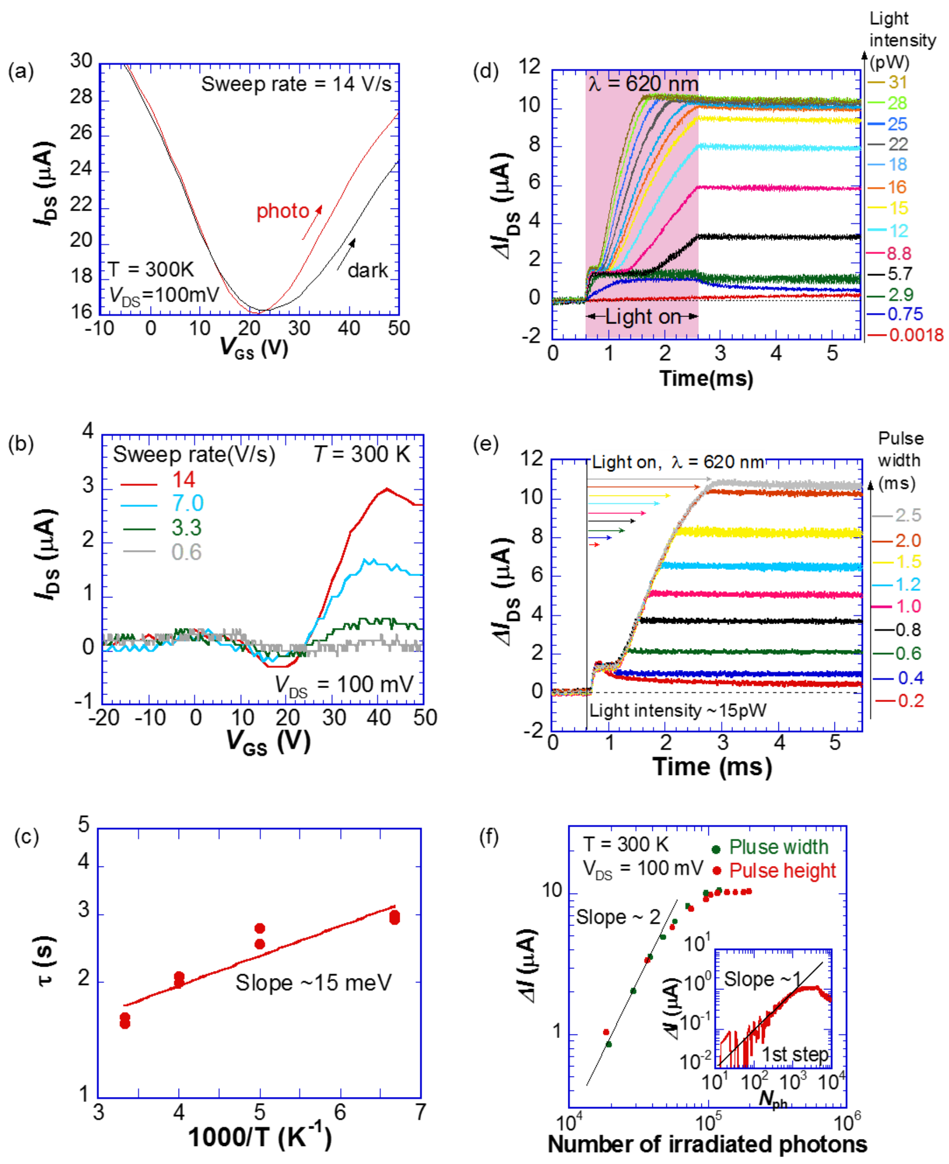
(a)
Sweep rate = 14 V/s
photo
dark
T = 300K
VDS=100mV
IDS (μA)
VGS (V)
(b)
Sweep rate(V/s)
T = 300 K
14
7.0
3.3
0.6
VDS = 100 mV
IDS (μA)
VGS (V)
(c)
Slope ~15 meV
τ (s)
1000/T (K-1)
(d)
λ = 620 nm
Light on
Light intensity (pW)
31
28
25
22
18
16
15
12
8.8
5.7
2.9
0.75
0.0018
ΔIDS (μA)
Time(ms)
(e)
Light on, λ = 620 nm
Light intensity ~15pW
Pulse width (ms)
2.5
2.0
1.5
1.2
1.0
0.8
0.6
0.4
0.2
ΔIDS (μA)
Time (ms)
(f)
T = 300 K
VDS = 100 mV
Pluse width
Pulse height
Slope ~ 2
Slope ~1
1st step
ΔI (μA)
Nph
Number of irradiated photons

Figure S8
